# Generation of multiparametric MRI maps by using Gd-labelled- RBCs reveals phenotypes and stages of murine prostate cancer

**DOI:** 10.1038/s41598-018-28926-5

**Published:** 2018-07-12

**Authors:** Giuseppe Ferrauto, Enza Di Gregorio, Stefania Lanzardo, Laura Ciolli, Manuela Iezzi, Silvio Aime

**Affiliations:** 1 Molecular Imaging Center, Department of Molecular Biotechnologies and health Sciences, University of Torino, Torino, Italy; 2 Department of Molecular Biotechnologies and health Sciences, University of Torino, Torino, Italy; 3 Department of Medicine and Aging Science, Center of Excellence on Aging and Translational Medicine (CeSi-Met), G. D’Annunzio University, Chieti-Pescara, Italy

## Abstract

Prostate Cancer (PCa) is the second most common and fifth cause of cancer-related mortality in males in Western Countries. The development of innovative tools for an early, more precise and noninvasive diagnosis is a medical need. Vascular volume (Vv) and hypoxia are two of the most important tumor hallmarks. Herein, they have been assessed in TRAMP mice by using MRI. Their quantification has been carried out by injecting autologous Red Blood Cells (RBCs), *ex vivo* labelled with Gd-HPDO3A or Gd-DOTP complexes, respectively. Gd-labelled-RBCs are stably confined in the intravascular space, also in presence of a very leaky tumor endothelium, thus representing efficient probes for vascular space analysis. Vv enhancement and hypoxia onset have been demonstrated to be present at early stages of PCa and their expression largely increases with tumor development. Moreover, also Diffusion weighted MRI and Amide Proton Transfer MRI have been herein applied to characterize PCa. The herein applied multiparametric MRI (mpMRI) analysis allows a detailed *in viv*o characterization of PCa, in which each histotype and cancer stage displays a specific MRI pattern. This provides an unprecedented opportunity to feature prostate tumor, making possible a non-invasive, precise and early diagnosis, which could direct treatments towards a more personalized medicine.

## Introduction

Prostate Cancer (PCa) is the second most common and fifth cause of cancer-related mortality in males in Western Countries^1^. As common for many solid tumors, a panel of hallmarks can be analyzed to characterize tumors and establish the proper phenotype. Firstly, during tumor growth, there is a large increase of the vasculature due to the onset of angiogenesis^2^. This is necessary to supply nutrient and oxygen to tumor cells, so sustaining their growth. Because of the so rapid tumor growth, often the increase of vascular network is not sufficient. In addition, intratumoral vascular network is not well organized, with a very chaotic architecture, the absence of organized shape and vessel distribution and an unregulated blood flow^3^. Altogether is responsible for the onset of hypoxia in PCa tissue.

For this reason, Vascular volume (Vv)^4,5^ and tumor hypoxia^6,7^ are considered two important PCa hallmarks. In addition, other hallmarks can be used to characterize tumors, namely prostate volume, cellularity^8^ and amide proton content^9^.

The access to the above reported set of biomarkers may be of great importance for (i) improving the accuracy of early PCa diagnosis, (ii) having a precise molecular tumor phenotyping, (iii) gaining new insights into the pathogenesis and the evolution of cancer and iv) helping the clinicians toward the best therapeutic treatment, specific for each patient (prostatectomy, radiotherapy or specific chemotherapy), thus avoiding overtreatment^1^.

For instance, it has been shown that PCa patients with evidence of hypoxia have a poor overall survival compared to patients with normoxia^10,11^.

Currently, the “*gold standard*” for tumor staging and molecular characterization relies on, as typical, the histological analysis of tissue obtained by ultrasound-guided prostate biopsies. This method has the important limitation of being invasive for patient, with the possibility of negative consequence of hemorrhage, inflammation and infection. This is exacerbated by the need of carrying out a multiple sampling to have information representative of the entire prostate tissue. Of note, 20% of prostate cancers are missed or undersampled during the biopsy session.

The use of diagnostic imaging techniques (MRI/Echography) is widely accepted in clinical practice, adding a good deal of information to the to classical stadiation exams (rectal examination). It allows adding significant incremental values for local staging, particularly in pre-operative identification of extraprostatic extension and seminal vesicle invasion^12,13^. Beyond this, diagnostic imaging techniques are currently not yet able to provide information on tumor biomolecular features in the same way of histology.

It is well-established that Magnetic Resonance Imaging (MRI) is a powerful tool to detect and characterize tumors since it allows obtaining images of soft tissues, with a very high spatio-temporal resolution, limited invasiveness and without using ionizing radiation^14^. This technique is highly versatile and, by properly designing the experimental protocol, may provide insights into several hallmarks that characterize tumors, among which Vv and hypoxia.

We have recently reported that an accurate assessment of Vv can be obtained by using *ex vivo* Gd-loaded-RBCs^15^ as the effect on T_1_ of water protons from the paramagnetic agents is limited to the vascular compartment in which RBCs reside. This approach appears an important step ahead in respect to the established indirect method by which the Vv is assessed upon measuring the relaxation enhancement of molecular Gd-containing contrast agents (CAs) that distribute in the vascular and extravascular compartments. Moreover, a further development of the use of Gd-labelled-RBCs has allowed the design of a pO_2_-responsive CAs that can report on the tumor hypoxia on the basis of the actual ratio between oxy- and deoxy-Hb in the labelled erythrocytes^16^.

Herein, we show how Gd-RBCs can be suitable for assessing Vv and hypoxia *in vivo* in murine models of PCa. The loading of Gd-complexes inside RBCs makes them suitable micro-devices for diagnostic imaging. The merging of information attained by using Gd-RBCs with those obtained with classical MRI experiments (T_2w_, DWI, APT) allows getting an improved characterization of PCa at different stages of development (early, middle and late) or with different histotypes (high grade intraepithelial-neoplasia(HGPIN), well differentiated androgen dependent Adenocarcinoma(WDADC), poorly differentiated neuroendocrine carcinoma(PD) PCa and phyllodes tumor).

The herein reported multiparametric MRI (mMRI) characterization has been carried out in transgenic Adenocarcinoma of Mouse Prostate (TRAMP) model^17^.

## Methods

### Chemicals

Gd-HPDO3A was kindly provided by Bracco Imaging S.p.A (Colleretto Giacosa, Torino, Italy) (chemical structure in Fig. S2). Gd-DOTP was synthesized as previously reported^16^ (chemical structure in Fig. S2). Ficoll Hystopaque, heparin and all other chemicals were purchased from Sigma-Aldrich Co. LLC.

### Animals

Transgenic Adenocarcinoma of Mouse Prostate (TRAMP)^17^ and C57BL/6 mice were kept in standard housing with rodent chow and water available ad libitum and a 12 h light/dark cycle. Experiments were performed according to the national and European (directive 2010/63) regulations and were approved by the local ethical committee for animal experiments. Male C57BL/6 mice were purchased from Charles River Laboratories (Calco, Italy) and used as healthy control. TRAMP mice were bred at the Department of Medicine and Aging Science, Center of Excellence on Aging and Translational Medicine (CeSi-Met), G. D’Annunzio University, Chieti-Pescara, Italy. Mice were maintained in specific pathogen-free conditions (Allentown Caging Equipment, Allentown).

Mice were analyzed at times corresponding to progressive stages of tumor development, namely (i) at the early stage (10–12 weeks), (ii) at the middle stage (16–20 weeks) and at the late stage (28–32 weeks). Healthy C57BL/6 mice were used as controls (n = 8 for the different groups).

For late stage tumor, three different phenotypes have been investigated, namely: (i) well differentiated adenocarcinoma (WDADC), (ii) poorly differentiated carcinoma (PD) and (iii) phyllodes tumor of seminal vesicles. The experimental set-up for images acquisition and histology validation is reported in Fig. S1A.

For the MRI and PAI experiments, mice were anesthetized by intramuscular injection of a mixture of Tiletamine/Zolazepam (Zoletil 100, Virbac, Milan, Italy) 20 mg/kg and xylazine (Rompun; Bayer, Milan, Italy) 5 mg/kg.

### Isolation of RBC and labeling by hypotonic swelling

Erythrocyte separation was carried out using the Ficoll Hystopaque^1039^ methodology^18^. Blood was obtained from syngeneic donor mice, diluted 1:1 with Phosphate Buffered Saline (PBS), stratified into Ficoll Hystopaque^1039^ and centrifuged for 30 minutes at 1500 rpm, 25 °C. The pellet (containing erythrocytes) was separated from the other components, washed three times with Heparin-supplemented PBS and centrifuged at 2300 rpm for 10 min and 4 °C. The separated erythrocytes were then used for the labeling experiments. The two Gd-complexes were loaded inside two pools of RBCs by using the osmotic swelling methodology^18,19^. A scheme of the hypotonic swelling procedure is reported in Fig. S2). Cells were placed in a hypotonic solution (160 mOsm/l) containing the Gd-complex to be loaded (Gd-HPDO3A 100 mM or Gd-DOTP 20 mM, respectively), at 4 °C for 30 min, under moderate stirring. Then, the isotonic condition (280 mOsm/l) was restored by adding a PBS buffer with an overall osmolarity of 400 mOsm/l (1:1 *vol:vol*). The difference in the concentrations of the two used Gd-complexes depends on the different net charge of the two complexes (5- for Gd-DOTP and 0 for Gd-HPDO3A, respectively) and thus on the different osmolarities of their solution^16,19^. The labeled erythrocytes were extensively washed with PBS to remove the non-internalized Gd-complexes. The osmolarities of the solutions were monitored using a manual Löser type 6 Micro-Osmometer. The amount of Hb was evaluated by measuring SORET band region absorbance (413 nm) using a 6715 UV-Vis Spectrophotometer Jenway (Bibby Scientific Limited, Beacon Road, Stone, Staffordshire, ST15 OSA, UK).

For assessing the amounts of internalized Gd-complexes, water was added to the RBC-containing suspension (50:1 *vol/vol*) to reach an external osmolarity <10 mOsm/l under vigorous stirring to facilitate cellular lyses. 1 ml of concentrated HNO_3_ (70%) was then added to 100 μl of each specimen and digested by microwave heating (Milestone MicroSYNTH, Microwave lab station equipped with an optical fiber temperature control and HPR-1000/6 M six position high-pressure reactor, Bergamo, Italy). After digestion, each sample was brought to a volume of 2 mL with ultrapure water, filtered on a 0.4 μm filter and analyzed by ICP-MS, using a Thermo Scientific ELEMENT 2 ICP-MS -Finnigan, Rodano (MI) to determine Fe and Gd concentrations (three replicates of each sample). The total amount of Gd-ions was divided for the number of RBCs in the specimen (as estimated by assessing the absorption intensity of SORET band) in order to obtain the number of Gd-complexes for RBC.

### MRI experiments and data analysis

MR images were acquired at 7.1 T on a Bruker Avance 300 spectrometer equipped with the Micro 2.5 microimaging probe at room temperature (R.T. = 21 °C).

High resolution T_2W_ images were acquired by using a standard RARE (Rapid Acquisition with Refocused Echoes) sequence with the following parameters: TR = 4000 ms, TE = 36 ms, RARE factor = 8, flip angle = 180°, number of averages = 8, FOV = 40 mm × 40 mm, slice thickness = 0.5 mm, matrix size 384 × 384, spatial resolution = 0.104 mm/pixel × 0.104 mm/pixel.

T_1W_ images were acquired by using a standard MSME (multislice multiecho) sequence with the following parameters: TR = 200 ms, TE = 3.4 ms, number of average = 6, FOV = 40 mm × 40 mm, slice thickness = 1 mm, matrix size 128 × 128, spatial resolution = 0.273 mm/pixel × 0.273 mm/pixel. In order to eliminate the flow artifact, axial saturation slices were applied close to the region of acquisition with the following parameters; slice thickness 20 mm, Hermitian shape pulse, length 1 ms, Flip Angle of 90°. A glass tube containing water as reference was inserted close to the mouse body. Three T_1W_ images were acquired for the assessment of Vv and hyp (Fig. S2). The first one was a pre-contrast image. The second one was acquired after the *i.v*. administration of c*a*. 1.5 × 10^9^Gd-HPDO3A-RBCs in the tail vein of mice. This dose corresponds to *ca*. 10% of the total number of naturally occurring RBCs in the body’s mouse^15^. In order to avoid hypervolemia, the administration of labeled-RBC was preceded by the withdrawal of an amount of blood which contained as many RBCs as the ones to be re-injected.

After the acquisition of these two images, the T_1_ contrast enhancement (T_1enh_%) was calculated as follows:

$${T}_{1enh}{\rm{ \% }}=\frac{S{I}_{post}-S{I}_{pre}}{S{I}_{pre}}\times 100$$

where SI_post_ and SI_pre_ are the signal intensities (both normalized by dividing for an external standard reference) *post* and *pre* the injection of Gd-HPDO3A-RBCs.

These images were used for the assessment of Vv, as previously reported^15^. Briefly, Regions of Interest (ROIs) were manually drawn inside a reference blood vessel and in the tumor region and T_1enh_ % was calculated in both the ROI as reported above. The ratio between T_1enh_ % measured in the tumor region and T_1enh_ % of the reference blood vessel yields the mean vascular volume in the tumor region^15^ (Fig. S3).

The third T_1W_ image was acquired after the injection of Gd-DOTP-RBCs^9^. T_1enh_ % was calculated as above reported, by using the post Gd-HPDO3A-RBCs T_1w_ image as “*pre”* and post Gd-DOTP-RBCs T_1w_ image as” *post”*. In this case, the ratio between T_1enh_ % of the tumor region and T_1enh_ % of the reference blood vessel allowed taking into account the vascular contribution (Fig. S4).

The images obtained upon Gd-DOTP-RBC and Gd-HPDO3A-RBC administration were combined in order to obtain normalized maps that report about the oxygenation state independently of the actual vessels’ density in the voxel. The ratio between the signal enhancement *post* Gd-HPDO3A-RBCs administration (R_GdHPDO3A_) and *post* Gd-DOTP-RBCs administration (R_1GdDOTP_) provides information about the oxygen content^16^. Owing to the longer R_1_ in the presence of deoxy-Hb, the rationing between R_1GdDOTP_ and R_1GdHPDO3A_ provides the deoxygenation index for each pixel:

$${\rm{Deoxygenation}}\,{\rm{Index}}\,({\rm{DI}})=\frac{{R}_{1GdDOTp}}{{R}_{1GdHPDO3A}}$$

Relative Oxygenation Index (OI%) was calculated as follows:

$$OI \% =(1-DI)\times 100$$

The Diffusion Weighted MR Images (DWI)^8^ were acquired by using a Spin Echo sequence with the following parameters: TE = 27 ms, TR = 1250 ms, number of average = 1, FOV = 35 mm × 35 mm, slice thickness = 1 mm, matrix size 128 × 128, spatial resolution = 0.273 mm/pixel × 0.273 mm/pixel. Three different B-values were used, *i.e*. 0, 500 and 1000 s/mm^2^. Apparent diffusion constant (ADC) maps were calculated fitting DW-MRI signal intensity as a function of B-values by using Bruker ParaVision 5.1 software. ADC mean values were calculated superimposing ADC maps on the selected ROIs.

The assessment of protein content in the tumor region was obtained by acquiring Amide Proton

Transfer (APT) –MR image^20–22^. For this purpose, Z-spectra were acquired in a range of ±200 ppm by acquiring a total of 121 data points (steps of 0.5 ppm in the range from 0 to ±10, steps of 1 ppm in the range from ±10 to ±20; steps of 2 ppm in the range from ±20 to ±200 ppm).

A typical RARE spin–echo sequence with an echo time of 3 ms and a TR value of 8 s was used. An isotropic 64 × 64 acquisition matrix with a FOV of 30 mm × 30 mm and a slice thickness of 1 mm was used. The whole sequence was preceded by a saturation scheme consisting of a continuous rectangular wave pulse 3 s long with a radiofrequency B_2_ intensity of 6 μT. The Z-spectra were analyzed by custom-made software, compiled in the Matlab platform. The Magnetization Transfer asymmetry (MTR_asym_) was calculated as follows:

$$MT{R}_{asym} \% =1-\frac{S{I}_{(\omega )}}{S{I}_{(-\omega )}}\times 100$$

where SI(ω) and SI(−ω) are the MR signal intensities in the Z-spectrum at 3.5 ppm and −3.5 ppm, respectively.

### Ultrasound and PhotoAcoustic Imaging

PhotoAcoustic (PA) images were acquired on a VisualSonics Vevo 2100 LAZR Imaging Station (VisualSonics, Inc., Toronto, Canada). Mouse’s hair was removed from areas of interest using a depilatory cream and Ultrasound gel was applied. Mice were excluded from PAI analysis when not homogenous dark regions were present in the skin^23,24^.

In order to collect anatomical information at high resolution, B-mode imaging was acquired using a high-frequency ultrasound probe (MS550D, VisualSonics, Canada, broadband frequency: 22 MHz–55 MHz, image axial resolution: 40 um) at 40 MHz. Oxygen saturation and Hemoglobin concentration were measured at 21 MHz frequency (LZ250, VisualSonics, Canada). Hb content was measured with a laser set at 812 nm^15,23^. Oxygen saturation (SO_2_%) was measured with PA dual-wavelength imaging at 750 and 850 nm^16,23^. All PA images were co-registered with grey scale B-mode images. The quantification of total hemoglobin and oxygen saturation was achieved using HemoMeaZure™ tool and OxyZated™ tool (VisualSonics, Canada), respectively.

PAI data for Hb content (at 812 nm) and for SO_2_ were correlated with MRI data for Vv and OI, respectively.

### Immunohistochemical and Immunofluorescence analysis

Mice were sacrificed by cervical dislocation. Prostate and seminal vesicles were excised for histological analysis. They were divided into two parts and washed twice in PBS (Sigma-Aldrich, Milano, Italy). One part was fixed in PLP (Paraformaldehyde/Lysine/Periodate) and mounted in Optimal Cutting Temperature (OCT) compound (Bio-Optica, Milan, Italy) for the immunofluorescent analysis. The second part was fixed in 10% Neutral Buffered Formalin (Bio-Optica, Milan, Italy) and then embedded in paraffin; 5 μm slides were cut and stained with Hematoxylin (BioOptica) and Eosin (BioOptica) for histological and immunohistochemical examination.

For the immunohistochemical study, slides were stained with rat anti-mouse CD-31 antibody (DIA-310, Dianova) at 1:20 diluition, for 30 minutes at R.T., followed by the appropriate secondary antibody. Immunoreactive antigens were detected using alkaline phosphatase conjugated streptavidin (Thermo Scientific) and Vulcan fast red chromogen (Biocare Medical). After chromogen incubation, slides were counterstained with Hematoxylin and images were acquired by Leica DMRD optical microscope (Leica). The vascularization was analyzed evaluating CD31+ endothelial cells on the digital images of 2–4 samples per group (3–5 × 100 microscopic fields per sample) with Adobe Photoshop by selecting vessels with the Magic Wand Tool and reporting the number of pixels indicated in the histogram window in respect to the total area. For immunofluorescence, 4–6 µm cryostat sections were air-dried, fixed in ice-cold acetone for 10 min and incubated with the following primary antibodies: rat monoclonal anti-CD31 (550274, BD Pharmingen) mixed with rat monoclonal anti-CD105 (550546, BD Pharmingen, San Diego, CA) and rabbit polyclonal anti-NG2 (ab5320, Millipore) followed by secondary antibodies conjugated with Alexa 546 and Alexa 488 (Invitrogen, Life Technologies, Monza, Italy), respectively. Nuclei were stained with DRAQ5 (Alexis, Life Technologies, Monza, Italy). Image acquisition was performed using Zeiss LSM 510 META confocal microscope. The vascularization was analyzed evaluating CD31 +/105+ endothelial cells and NG2+ pericytes on digital images of 2–4 samples per group (2–9 × 40 microscopic fields per sample) with Adobe Photoshop by selecting vessels (red pixels) and pericytes (green pixels) with the Magic Wand Tool and reporting the number of pixels indicated in the histogram window as percentage of green pixels on red pixels.

### Statistical Analysis

Data are represented as mean ± SD. A paired two-tail Student’s t-test was used for all the experiments.

## Results

### High resolution T_2w_ MR Images

**Figure 1:**
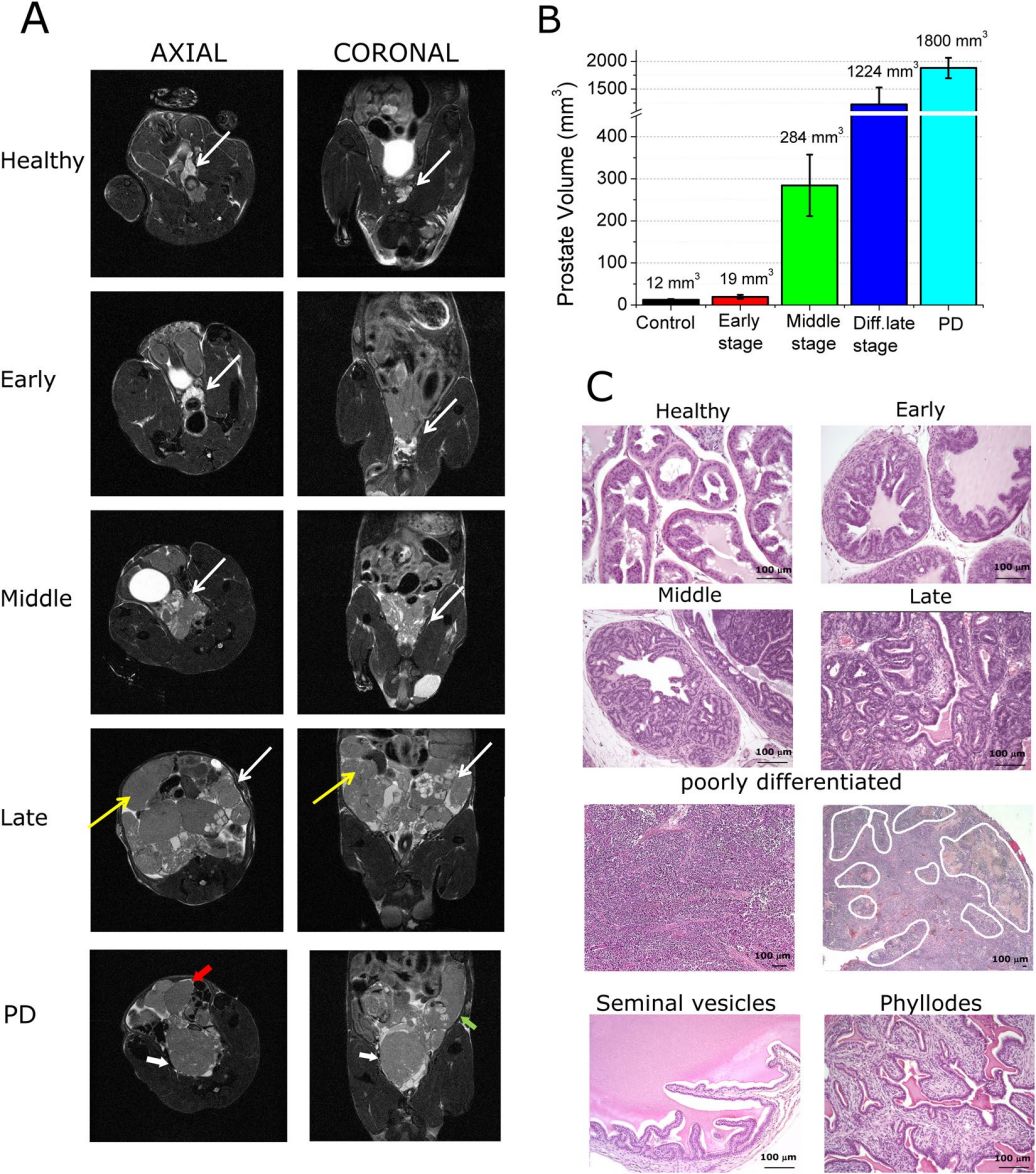
(**A**) Axial (*left column*) and Coronal (*right column*) high resolution T_2w_ MR images of a representative healthy control C57BL/6 mouse (*first row*) and representative TRAMP mice at three development stages namely: early stage (*second row*), middle stage (*third row*), differentiated late stage (*fourth row*) (Thin white arrow indicates prostate, thin yellow arrow indicates tumoral lesion in seminal vesicles, *i.e*. phyllodes tumor). (*fifth row*) Poorly differentiated late stage, PD (thick white arrow = poorly differentiated PCa region, red arrow = differentiated PCa, green arrow = invaded seminal vesicles); (**B**) Histograms reporting prostate’s size for the investigated groups. Data are reported as mean ± SD; (**C**) Representative H/E histological images of prostate tissue in healthy and TRAMP mice and of healthy seminal vesicles and phyllodes tumour (Magnification 200X. Poorly differentiated tumor image on the left: magnification 100X. Poorly differentiated tumor image on the right: magnification 25X). White outlines indicate areas of necrosis. PD = poorly differentiated. (n = 8).

High resolution axial and coronal T_2w_ MR images at 7.1 T (without the administration of CAs) provide an accurate anatomical definition of the tumor region (Fig. 1A). Identification of prostate in MR images of mice has been carried out as previously reported^25^. A representative scheme of anatomy of mouse prostate has been reported in Fig. S1B, whereas Fig. S1C report two representative T_2w_ MR images of murine prostate.

At the early stage (Fig. 1A *second row*), upon comparing to control healthy mice (Fig. 1A *first row*), one may note that there is only a slight prostate enlargement without any detectable seminal vesicle involvement (*prostate is indicated by thin white arrows*).

Conversely, in the middle and late stages, there is a marked increase of the prostate size (Fig. 1A *third and fourth row, respectively*) (*prostate is indicated by thin white arrows*). At late stage, Tumor lesion is present also in the seminal vesicles, with the typical phyllodes structure (Fig. 1A *fourth row, thin yellow arrows*).

In poorly differentiated (PD) PCa (Fig. 1A *fifth row)* two distinct and evident areas are detectable, namely poorly differentiated and differentiated PCa regions *(Thick white arrows indicates PD tumor region, red arrow indicates differentiated tumor region and green arrow indicates phyllodes tumor inside seminal vesicles*).

Prostate size has been measured from MR images (Fig. 1B) and it is higher in presence of cancer than in control healthy tissue. At the early stage the volume increase is *ca*. 60%. The prostate widens 20-fold and >100-fold in PCa at middle and late stages, respectively (Fig. 1B).

It is worth of note that in the case of control as well for early and middle stages of TRAMP mice, the prostate region is well distinguishable from the seminal vesicles since the latter structures appear hypointense. On the contrary, at the late stage the two anatomical structures are not because the seminal vesicles are filled by cancer cells thus the signal intensity is close to the one of prostate tissue. Thus, it is not possible to distinguish from prostate tumor and phyllodes tumor. Prostate in mice with poorly differentiated (PD) tumor is bigger than the one of mice with well differentiated Adenocarcinoma (WDADC) (1800 mm^3^ *vs*. 1224 mm^3^, corresponding to a 100-fold and 150-fold enlargement of prostate respect to control, respectively).

The tumor progression has been followed and characterized by histology. Histological H/E images of representative prostate of early, middle and late stages have been reported and compared with healthy control (Fig. 1C *first and second line*). The histological features of phyllodes like tumors are also compared to that one of healthy seminal vesicles.

Histologic analysis showed the prostate tissue in TRAMP mice was composed of pre-neoplastic and neoplastic lesions with distinct histotypes.

Normal mouse prostate was composed of small acini and ducts lined by cuboidal or columnar epithelium with moderate infolding and occasional tufting embedded in a loose connective tissue with few stromal cells and collagen fibers.

Prostate glands of early stage TRAMP mice presented high grade prostate intraepithelial neoplasia (HGPIN) occupying almost half of their area. HGPIN is composed of dysplastic columnar epithelial cells, with hyperchromatic nuclei, a few secretory vacuoles and a reduced nucleus–cytoplasm ratio. At this age HGPIN cells form papillae that project into the lumen.

Prostate glands from middle stage TRAMP mice were composed by ducts enlarged by prominent HGPIN lesions with focal zones of WDADC. At this age HGPIN features are more pronounced, the papillae are larger and occasionally merge forming pseudolumina. WDADC cells are arranged in abnormal glands or multiple papillae with vessel-rich stromal axes. Focally the tendency to form gland-like structures is lost, and the cells grow in strings or mats. WDADC zones display more mitoses than HGPIN. The stromal connective tissue surrounding ducts is also thickened and displays an increased cellularity.

Prostate glands from late stages mice are mainly occupied by well and moderately differentiated adenocarcinoma.

PD tumors are composed of diffusely growing small cells with a low nucleus–cytoplasm ratio (Fig. 1C *third line*). The papillary or gland architectural arrangement is completely absent. Mitotic figures are numerous and paralleled by apoptotic ones. Necrotic foci are also very frequent and evenly distributed in larger tumors. The analysis of low magnification H/E slices of PD tumors show presence of necrotic area (white ROIs in Fig. 1C *third line right*).

At middle and late PCa stage, seminal vesicles develop epithelial-stromal neoplasms resembling phyllodes tumors of prostate and seminal vesicles of humans. These epithelial-stromal neoplasms are composed of proliferative cellular spindled mesenchymal cells, with high nuclear to cytoplasm ratio, growing beneath the cuboidal to columnar epithelium. Frequently, also the epithelium proliferates forming papillary and tubuloglandular structure. These phyllodes like tumors completely fill the seminal vesicles forming large masses (Fig. 1C *fourth line*).

### Vascular Volume and Hypoxia

Vascular Volume (Vv) and hypoxia have been quantified in PCa TRAMP mice by using a recently reported contrast enhanced (CE)-MRI procedure based on the use of Red Blood Cells *ex vivo* labelled by hypotonic swelling with two Gd-complexes, namely Gd-HPDO3A and Gd-DOTP(Gd-RBCs) (Figs S2 and S3)^15,16^. This procedure allows entrapping high amounts of Gd-HPDO3A inside cells (*ca*. 3 × 10^10^ Gd-complex/cell) without recording any detectable toxic effect^15^.

**Figure 2:**
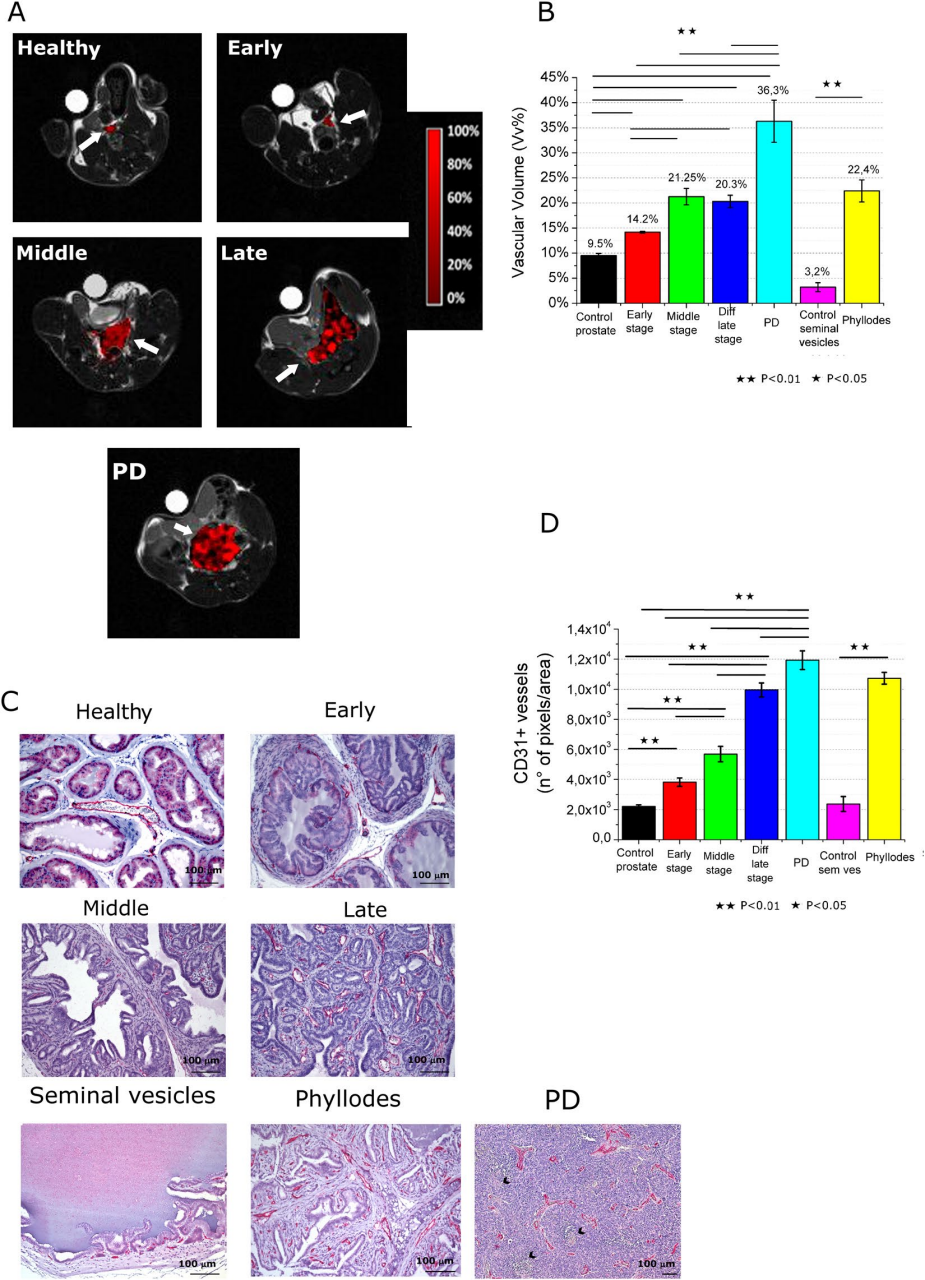
(**A**) Representative Vascular volume maps of representative healthy C57BL/6 mouse and TRAMP mice at early, middle and late development stages (including PD tumor); (**B**) Mean Vascular volume in the prostate region for the different groups and control health mice. Data are reported as mean ± SE; (**C**) CD-31 immunohistochemistry (*in red*) of representative healthy C57BL/6 mouse and TRAMP mice prostate and of healthy seminal vesicles and phyllodes tumor (Magnification 200×, black arrows indicate necrosis); (**D**) Quantification of vessels by image analysis of red pixels (CD-31 staining). (n = 8).

In Fig. 2A, representative axial slices of Vv maps of TRAMP mice are reported and compared to healthy control. Mean Vv of the entire prostate region have been calculated and reported in Fig. 2B. Mean prostate Vv in healthy mice is 9.5%. This values increases in TRAMP mice at early and middle stage by reaching a value of 14.2% and 21.25%, respectively. For late WDADC stage the Vv is 20.3% indicating that there is no significant change of Vv in respect to the middle stage. In PD PCa (Fig. 2A *third line*) the vasculature appears quite heterogeneous with the presence of highly vascularized (*red pixels*) and unvascularized (*black pixels*) regions. In this histotype the Vv value is tremendously higher than in the other ones (Vv = 36.3%).

An analogue consideration can be done for phyllodes tumor, where there is large increase of Vv (Vv = 22.4%) respect to control health seminal vesicles (Vv = 3.2%), with a Vv value quite similar to that of WDADC late tumor (Fig. 2B).

Changes in vascularity have also been assessed by immunohistochemistry by means of anti CD-31 staining (Fig. 2C). During prostate cancer transformation, new vessels grow along the fibrovascular axes of neoplastic papillary structures. The prostate vessels’ density is already higher in respect to control healthy tissue since the first appearance of HGPIN. Furthermore, it increases during tumor growth as assessed by quantification of CD-31 positive pixels in images (Fig. 2D). In the case of PD tumors, the immunohistochemical staining for CD31 (Fig. 2C middle) showed in the non-necrotic areas the presence of a rich vascular network composed of numerous vessel of large, medium and small caliber. MRI assessment of Vv and CD-31 staining maintain an analogue trend for the different specimens (Fig. 2D).

In analogy to what reported by MRI, in phyllodes tumor the Vv is comparable to the one of late stage tumor.

Next, the Gd-labelled-RBCs based procedure has been employed for the assessment of the oxygenation level of the tumor region^16^. For this purpose, RBCs have been *ex vivo* labeled by hypotonic swelling in the presence of Gd-DOTP.

**Figure 3:**
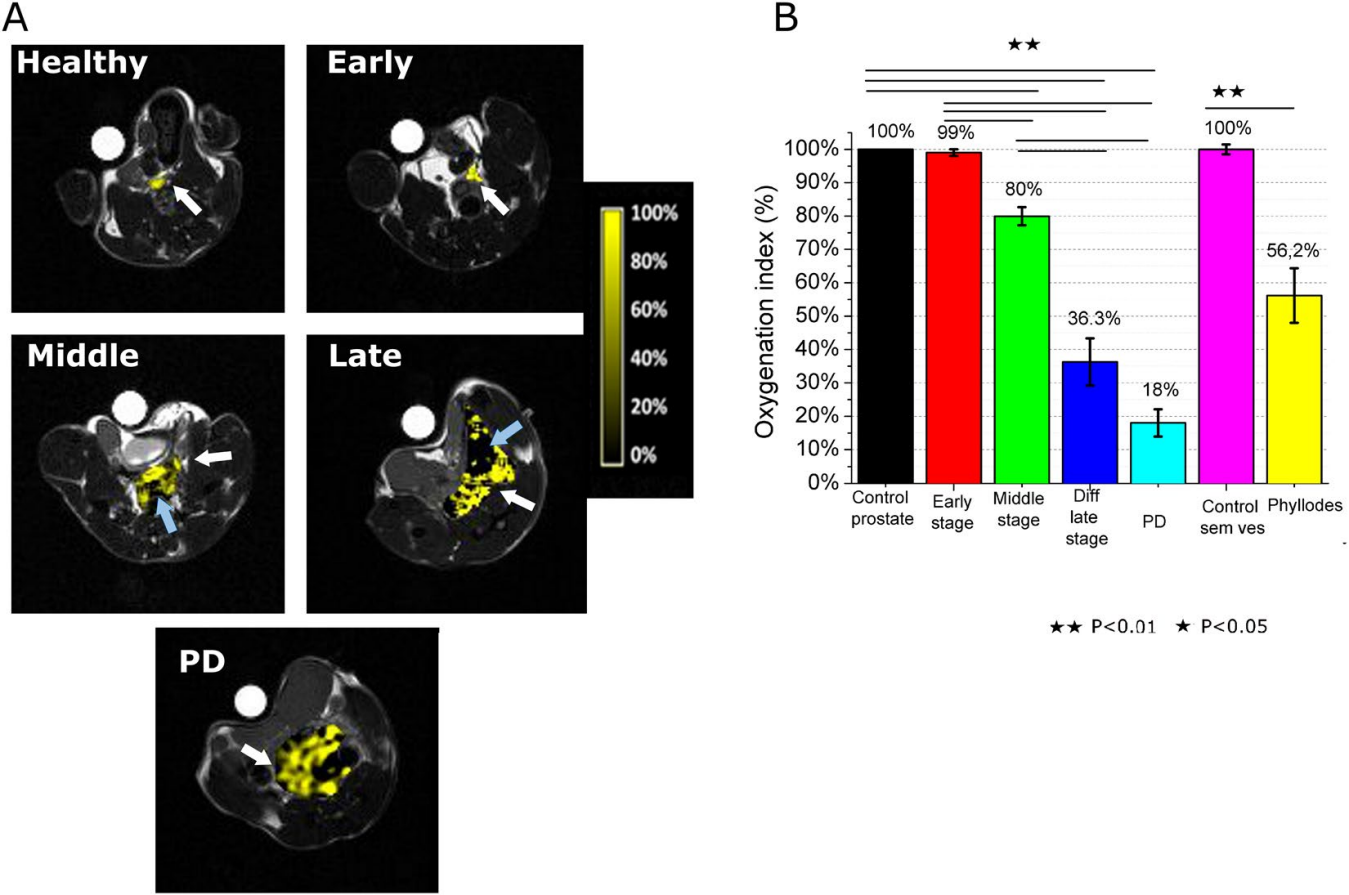
(**A**) Relative oxygenation maps of representative healthy C57BL/6 mouse and TRAMP mice; (**B**) Relative oxygenation index in the prostate region for the different groups and control mice. Data are reported as mean ± SE. (n = 8).

In Fig. 3A,B representative axial slices of oxygenation maps of TRAMP mice at the three tumor growth stages have been reported and compared to healthy control mice, for which the Oxygenation index is set equal to 100%.

In Fig. 3A, oxygenated regions are represented by yellow pixels, whereas hypoxic regions are represented by black pixels. At the early stage of tumor progression, the prostate region still shows the same oxygenation index of the control. On passing from the early to the middle and WDADC late stage, the OI% decreases first to 80% and then it drops to 36.3%, respectively (Fig. 3B). This severity is exacerbated in PD tumors in which OI goes down to 18.3%, with the presence of a highly hypoxic core region (Fig. 3A *third line*).

The presence of highly hypoxic regions at middle and late stages is indicated by the light blue arrows in Fig. 3A. In phyllodes tumors the hypoxia is less severe than in differentiated late stage PCa (OI = 56.2%).

Both Vascular volume and oxygenation data have been validated *in vivo* by using Photoacoustic Imaging (Fig. S5)^23,24^. It shows an increase of Vv and a decrease of oxygen content on going from early to late stage. Data obtained by MRI display a good linear correlation with those obtained by PAI (Fig. S5).

### Diffusion Weighted Imaging (DWI)-MRI

**Figure 4:**
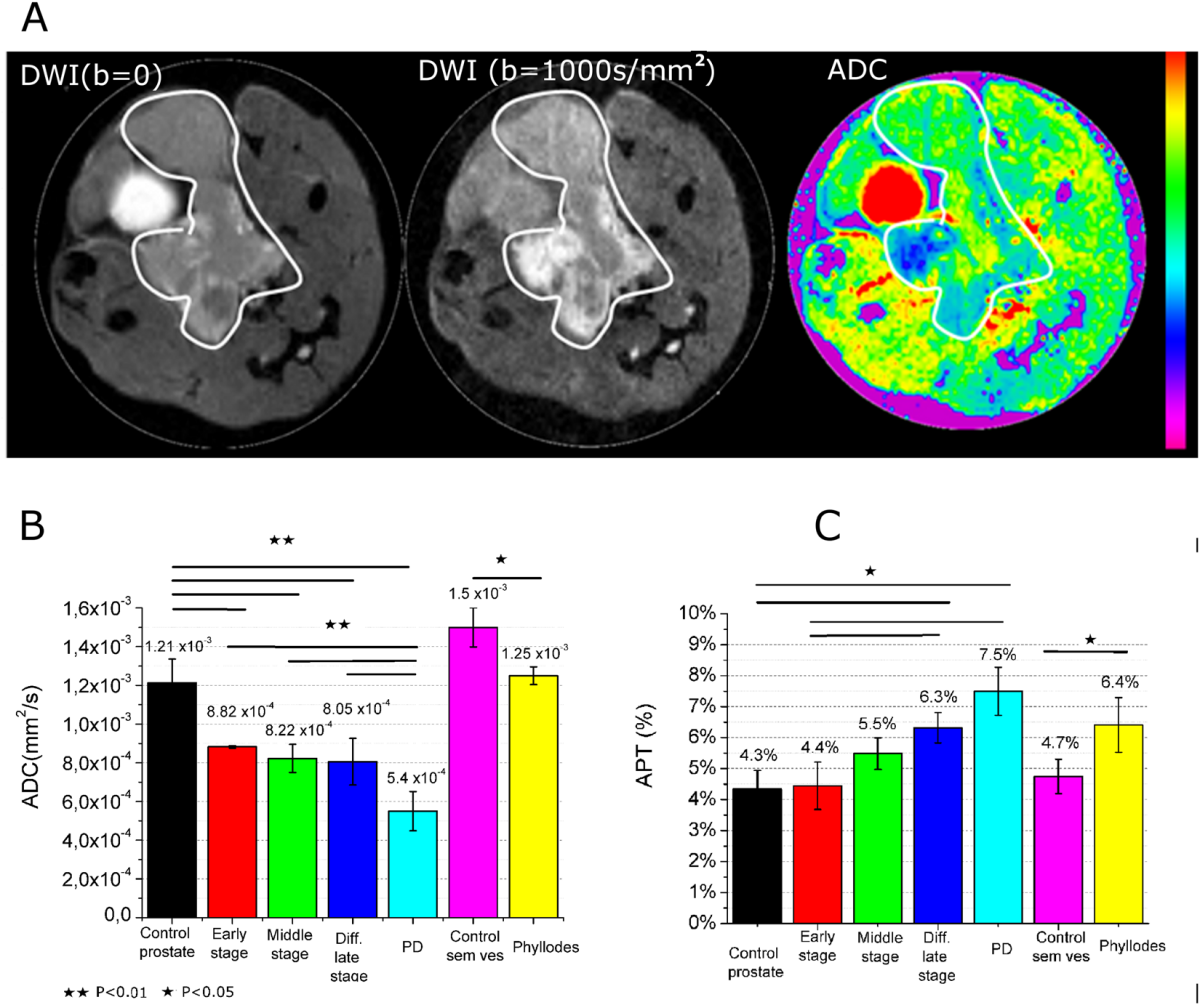
(**A**) Representative DW image (b = 0 and 1000 s/mm^2^ for left and middle images, respectively) and ADC weighted image of a TRAMP mouse at middle stage; (**B**) Apparent Diffusion coefficient (ADC) value in the prostate region for TRAMP and heathy control mice; (**C**) Magnetization Transfer Ratio (MTR) % value in the prostate region for TRAMP and healthy mice. Data are reported as mean ± SE. (n = 8).

PCa at the three stages have also been characterized by means of DWI-MRI for the acquisition of Apparent Diffusion Coefficient (ADC) map^8^. Representative images of axial slice of PCa tissue in TRAMP at late stage of tumor has been reported in Fig. 4A (DW images with b-value = 0 or 1000 s/mm^2^ and ADC-weighted images). Images of prostate tissue in TRAMP mice at the three tumor stages and control healthy mice have been reported in Fig. S6.

Water mobility in PCa is lower than in healthy prostate. ADC images provide additional morphological information in respect to T_2w_ images. In fact, in T_2w_ images there is continuity between prostate cancer tissue and seminal vesicles. Conversely, by looking at the ADC images it is possible to notice a clear difference between these two tissues because ADC values in the prostate tissue are lower than in seminal vesicles. Moreover, there is a gradient in the seminal vesicles ADC value that is lower in proximity of the prostate tissue (Fig. 4A).

The mean ADC values for the three stages of PCa development have been obtained and compared with healthy control mice (Fig. 4B). This value is 1.2 ± 0.1 × 10^−3^ mm^2^/s in control healthy mice and reduces to 8.8 ± 0.1 × 10^−4^, 8.2 ± 0.7 × 10^−4^ and 8.0 ± 1 × 10^−4^ mm^2^/s for the three PCa tumor stages, respectively. In PD PCa, ADC value is much lower than the one reported for the other histotypes (5.5 ± 0.1 × 10^−4^ mm^2^/s). In the phyllodes tumor of seminal vesicles, ADC value is slightly than that of healthy seminal vesicles (ADC = 1.25 ± 0.05 × 10^−3^ mm^2^/s and 1.5 ± 0.1 × 10^−3^ mm^2^/s, respectively), thus showing also a higher cellularity of this tumor phenotype respect to healthy tissue.

### Amide Proton Transfer (APT) imaging

PCa MRI characterization has been complemented with the acquisition of Amide Proton Transfer (APT) MRI^9,20–22^ (Figs 4C and S7). No significant difference has been noted between control healthy mice and TRAMP mice at the early stage (4.3% *vs* 4.4%).

A slight (5.5%) and a marked (6.3%) increase of APT is present for middle and late PCa, respectively. In PD PCa, APT value increases up to 7.5%. In phyllodes tumor it is 6.4%.

All reported data for the different phenotypes have been summarized in Table 1.

**Table 1:** Multiparametric characterization of TRAMP mice and comparison with health C57 mice. [* It is not possible to distinguish by T_2w_-MRI prostate tumor from phyllodes tumor].

	Control (*ref*.)	Early stage	Middle stage	Late stage*		
				WDADC	Phyllodes	PD
Mouse age (weeks)	8–32	8–14	20–24	28–32		
Prostate Volume (mm^3^)	12 ± 2	19 ± 5	284 ± 73	1224 ± 305		1800 ± 206
Vv %	9.5	14.2	21.25	20.3	22.4	36.3
OI %	100	99	80	36.3	56.2	18.3
ADC (mm^2^/s)	1.2 ± 0.1 × 10^−3^	8.8 ± 0.1 × 10^−4^	8.2 ± 0.7 × 10^−4^	8 ± 1 × 10^−4^	1.25 ± 0.05 × 10^−3^	5.5 ± 0.1 × 10^−4^
APT %	4.3	4.4	5.5	6.3	6.5	7.5
Remarks		- Small increase of prostate size -Moderate increase of Vv -No O_2_ reduction - Reduction of ADC value -No increase of APT signal	-Marked increase of prostate size -Marked Vv increase -Moderate O_2_ reduction - Reduction of ADC value -Moderate increase of APT signal	-Dramatic increase of prostate size -Large Vv increase -High O_2_ reduction -Reduction of ADC value -Increase of APT signal	-No change in ADC value	-Extremely large increase of prostate size -Very large Vv increase -Marked O_2_ reduction - High reduction of the ADC value -Marked increase of APT signal

### Histological characterization of vessels

Variations occurring in Vv, OI, ADC and APT could be strongly related to alterations in vessel permeability and structure.

**Figure 5:**
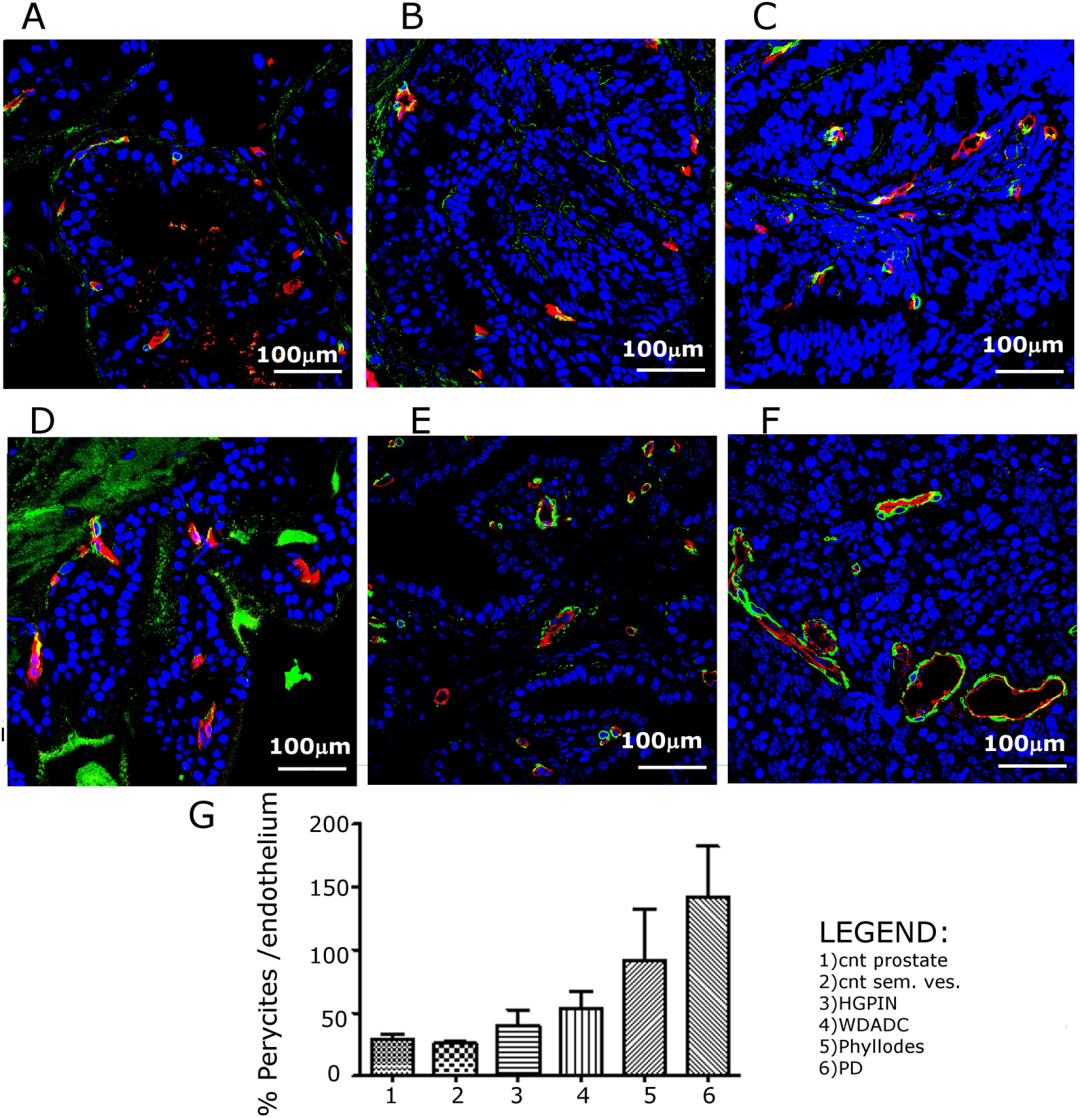
Representative CD-31/CD105 (*red*) and NG2 (*green*) immunofluorescence of healthy C57BL/6 mouse prostate (**A**) and seminal vesicle (**D**) and TRAMP mice prostate with HGPIN (**B**), WDADC (**C**) phyllodes (**E**) and PD (**F**) (Magnification 400×); (**G**) Quantification of the extent of pericytes presence on vessels. (n = 3).

Therefore, normal and neoplastic prostate and seminal vesicles have been characterized by immunofluorescence coupling the staining for young pericytes (anti-NG2) and endothelial cells (anti- CD31 and anti- CD105). In normal prostate and seminal vesicles, the small vessels localized within the ductule stroma are capillaries almost devoid of pericytes (Fig. 5A and D, respectively).

Larger diameter venules and arterioles covered with pericytes are present in the connective tissue around the ductules (Fig. 5A,D). During tumor progression both in prostate and seminal vesicle, ductule capillaries extended with the deeply invaginated layers of the epithelium progressively loosing normal organization and showing increasing number of young pericytes (Fig. 5B,C,E). In the PD tumor blood vessels lost the normal hierarchy, were heterogeneous in size and shape and showed an increased coverage of young pericytes both on large and small vessels (Fig. 5F).

## Discussion

Prostate cancer is the second most common cancer and the fifth leading cause of death from cancer in men (Globocan 2012-IARC)^1^. In the past years the increased awareness of the importance of cancer early diagnosis and the implementation of screening programs lead to an increase in low-risk forms of prostate cancer diagnosis and their overtreatment. As a matter of fact many of these lesions have a low biological potential and rarely progresses or causes harm if left alone. Despite this indolent behavior, these tumors are often treated with radiation or surgery, generating morbidities of treatment (*eg*, sexual, urinary, and gastrointestinal side effects, in about 15–20% of patients), increased risk of secondary malignancies (with radiation), and increased cost. Therefore, a detailed noninvasive imaging characterization of the tumor mass is of course very useful for screening patients and selecting the most proper personalized treatment to be applied (active surveillance, radiotherapy, chemiotherapy, surgery)^12,13,26,27^.

It is well-established that Magnetic Resonance Imaging (MRI) is the election technique to noninvasively image deep soft tissues, like prostate. mpMRI is able not only to detect and stage the extent of the disease but also to distinguish distinct hystotypes with different aggressiveness and clinical progression.

In this work, TRAMP mice^17^ have been used because they specifically express the oncogene SV40 T antigen (TAg) in the prostate epitelium thus generating a well localized cancer disease with a 100% of penetrance. Therefore, this model appears to be highly reproducible and closely mirror the pathogenesis of human PCa. In fact, these mice uniformly and spontaneously develop orthotopic PCa with well characteristic histological features, *i.e*. an initial prostate intraepithelial neoplasia (PIN) that evolves in well differentiated adenocarcinoma (WDAC), then in moderately differentiated and finally in poorly differentiated (PD) neuroendocrine carcinoma. In addition, also phyllodes tumors is present in TRAMP mice. As control, healthy C57BL/6 mice have been used. Healthy mice of different ages have been studied in order check whether differences in the analyzed parameters are present. No significant difference in the prostate features (size, vascular volume, hypoxia, water diffusion and protein content) has been recorded (*data not shown*).

TRAMP mice have been analyzed at three stages of tumor development, namely i) early stage (10–12 weeks), ii) middle (16–20 weeks) and iii) late stage (28–32 weeks). In the case of the late stage, three phenotypes have been compared, namely WDADC, PD and phyllodes tumor. In fact, a small population of TRAMP mice is known to develop a very aggressive PCa phenotype, characterized by the presence of poorly differentiated cancer cells (PD PCa) and by a very rapid tumor growth.

Mice have been subjected to mpMRI analysis and, successively, to *in vivo* Ultrasound/Photoacoustic(US/PAI) imaging and *ex vivo* histology to validate MRI results.

The extensive mpMRI characterization has been carried out with the aim of assessing i) tumor size, morphological feature, invasion of extraprostatic tissues and metastases presence (high resolution T_2w_ MRI), ii) increase of the tumor vascular volume and onset of hypoxia (T_1w_ MRI post administration of Gd-HPDO3A- and Gd-DOTP-labelled- RBCs), iii) changes in the tumor cellularity (by DWI- MRI) and in protein content in the tumor region (by APT-MRI).

MRI T_2w_ images showed that the prostate volume increases in PCa, especially at the middle and late stage (up to 150-fold in the case of PD phenotype). At the same time, seminal vesicles are characterized by the presence of tumor cells constituting phyllodes tumor. The tumor morphology assessed by MRI has been double-checked by US images and histology.

The increase of the prostate size is accompanied by a change in the vascular network.

Insights into the Vascular Volume and hypoxia onset have been gained by using autologous RBCs, *ex vivo* labelled with Gd-HPDO3A and Gd-DOTP, respectively^15,16,18–20^ (Fig. S2).

This has been obtained by placed RBCs in a hypotonic solution containing the paramagnetic complex to be loaded. Because of the osmotic difference between the intra- and the extra-cellular compartments, cells swell, increase size and enhance membrane permeability. Gd-complexes enter the cells moved by the concentration gradient. Extensive washing with fresh PBS ensures that not internalized Gd-complexes are eliminated. Gd-labelled- RBCs are then *i.v*. administrated to mice and they distribute in the whole vascular compartment, inside which they are fully retained for weeks. As previously reported, the MRI Signal Enhancement upon administration of Gd-HPDO3A-RBCs is proportional to the Vv in the Region of Interest(ROI). The comparison of MR images *pre* and *post* injection of Gd-HPDO3A-RBCs is therefore a direct reporter for changes in the Vv^15^ (Fig. S3). However, the quantitative assessment of the increase in the Vv, has been obtained by applying a ratiometric approach consisting in the comparison of the signal intensity observed in the tumor region in respect to the corresponding SI observed for voxels belonging to the large vessels^15^.

An analogue approach has been used for the assessment of the tumor hypoxia by loading Gd-DOTP inside RBCs. This complex has been demonstrated to be able to bind hemoglobin with a different affinity toward the oxy and the deoxy state (it binds 5-times more efficiently deoxy-Hb than oxy-Hb)^16^. When bound to Hb, its relaxivity is markedly higher in respect to the one of the free complex (37.5 mM^−1^s^−1^ for the deoxy-Hb, 22.9 mM^−1^s^−1^ for oxy-Hb and 4.8 mM^−1^s^−1^ for free complex)^16^. A ratiometric method has been set-up, thanks to which it is possible to extract quantitative information into the actual oxygenation level in the corresponding MR tumor images. Upon administration of Gd-DOTP-RBCs, the MRI Signal enhancement is determined both by the vascular volume and by the relative oxygenation level in Region of Interest (ROI)^16^. By combining the T_1w_-MR Images obtained after the administration of Gd-HPDO3A-RBCs (reporters of Vv) with those acquired in the presence of Gd-DOTP-RBCs (reporters of pO_2_) it is possible to extract quantitative information about the hypoxia level. The higher the amount of deoxyHb in the ROI the higher the Signal Enhancement in T_1w_-MR Images (Fig. S4).

In this work, both Vv and hypoxia have been assessed in TRAMP and healthy control mice and these biomarkers appear to be different for the various phenotypes.

In the presence of the tumor, the prostate tissue appears to be markedly more vascularized in respect to the healthy tissue. During the progression of the tumor, the vascular volume doubles. It is worth of note that there is no difference in the Vv value at the middle and late (WDADC) stage. This behavior may be accounted in term of the fact that the tumor mass increases too rapidly and the vascular system is not able to sustain this growth. At the early stage, the growth of the tumor vascular system is enough to provide O_2_ to cancer cells, thus hypoxia is not present. Conversely, at the middle stage, even in the presence of a large Vv, the vessels appear to be not properly functional. The architecture of the vascular system at this stage is chaotic, with the loss of organization and functionality of vessels^5–8,26,27^. This reduces the O_2_ supply to cancer cells thus triggering the onset of hypoxia. Finally, at the late stage, both the loss of vessel functionality and the insufficient growth of cancer vascular system leads to severe hypoxia^5–8^. These parameters are quite similar or even lighter in phyllodes tumors and much heavier in PD tumors. In *vivo* Photoacoustic (PA) data and *ex vivo* histological evidences corroborated the conclusions drawn by MRI. PA imaging allows detecting the presence of hemoglobin because this macromolecule absorbs light and releases energy that can be detected as Ultrasound waves (light to mechanical energy conversion). The acquisition of the PA signal upon irradiation at 812 nm wavelength provides quantitative information on the overall Hb content in the voxels of interest. The ratiometric analysis of the PAI signals upon irradiation at 750 nm and 805 nm wavelengths provides information into the relative oxygen content. A linear correlation between Vv assessed by MRI and PA signal intensity at 812 nm is observed (R^2^ = 0.989) (Fig. S5A).

Analogously a linear correlation is present between oxygenation index as assessed by MRI and oxygen saturation as assessed by PA (R^2^ = 0.97) (Fig. S5B).

After the *in vivo* imaging, prostates have been excised and histology has been carried out (Figs 1C, 2C and 5). Results appeared to be in line with what observed by MRI, with a progressive increase of tumor region in the prostate at the different stages and an overall enhancement of Vv, characterized by a loss of functionality at later stages.

Finally, mpMRI have been concluded by acquiring DWI and APT MR images.

DWI-MRI allows obtaining insights about the mobility of water molecules in the tissue thus providing an indirect information about cells density in the tumor region and, consequently, about the tumor growth and spread-out^8^. The growth of PCa introduces a change in the cellular organization in the prostate tissue causing an overall decrease of the water mobility that is well detected in the DWI-MRI modality. It has to be noted that the reduction of the ADC value is present at the early stage of tumor and it remains constant by passing to the middle and to the late stages. This reflects the fact that the early stage is characterized by the hyperplasia (thus higher cell content). The middle and late stages are characterized by dysplasia instead of hyperplasia. A further significant ADC reduction is present in PD tumors (up to 5.5 × 10^−4^ mm^2^/s).

Amide Proton Transfer (APT) MRI concluded the PCa characterization^9,20–22^. This technique detects endogenous mobile proteins and peptides in tissue *via* saturation of the amide protons of the peptide bonds. In such a way, APT provides insights into the tissue protein content, mainly in the intracellular compartment. The APT signal has been assessed by applying the asymmetrical analysis. A slight increase of APT signal is present on going from healthy mice to late stage TRAMP mice. The APT signal changes can be accounted in terms of i) an increase of intracellular protein content and ii) a variation of intracellular pH^9,23^. Although we cannot establish which the determinant of the observed changes is, it is evident that the measured APT parameter may be a useful complement to the mpMRI characterization of PCa.

At the end, Table 1 summarizes the tumor hallmarks obtained from MR images that outline specific features of each PCa stages/phenotype. The early stage is essentially characterized by (i) increase of prostate size, (ii) moderate increase of Vv and (iii) decrease of water mobility. The passage from early to middle stage is characterized by: (i) marked increase of prostate size, (ii) increase of Vv, (iii) moderate reduction of O_2_ supply, (iv) small increase of APT signal. Finally, the passage from middle to late stage is characterized by: (i) high increase of prostate size, (ii) severe reduction of O_2_ supply, (iii) increase of APT signal.

By concluding, different phenotypes and tumor stages can be assessed by using mpMRI. The administration of Gd-labelled-RBCs, that stably remain in the intravascular compartment, appears to be a powerful tool for the *in vivo* accurate assessment of Vv and hypoxia in the tumor region. One may expect that these findings may help clinicians in choosing the most suitable therapy, personalized for each patient, to select the group of men for whom radiation or surgery are appropriate therapeutic strategy, as well as for monitoring the therapeutic treatment outcome.

## Electronic supplementary material

Supplementary Information

SUPPLEMENTARY INFO with changes marked

1-Healthy axial.avi

2-Healthy coronal.avi

3- Healthy sagittal.avi

4-Early axial.avi

5-Early coronal.avi

6-Early sagittal.avi

7-Middle axial.avi

8-Middle coronal.avi

9-Middle sagittal.avi

10-Late axial.avi

11-Late coronal.avi

12-Late sagittal.avi

13-Undifferentiated coronal.avi

14-Undifferentiated sagittal.avi
