# Supplementary material for: Generation of multiparametric MRI maps by using Gd-labelled- RBCs reveals phenotypes and stages of murine prostate cancer
Source: Sci Rep. 2018 Jul 12;8:10567. doi: 10.1038/s41598-018-28926-5 (PMC6043588; doi:10.1038/s41598-018-28926-5)
Supplement: Supplementary file 1 — Supplementary Information [file 41598_2018_28926_MOESM1_ESM.docx]

**Supplementary materials for**

Generation of multiparametric MRI maps by using Gd-labelled- RBCs reveals phenotypes and stages of murine prostate cancer

Giuseppe Ferrauto^1†*^, Enza Di Gregorio^1†^, Stefania Lanzardo^2^, Laura Ciolli^3^, Manuela Iezzi^3^, Silvio Aime^1^

^1^ Molecular Imaging Center, Department of Molecular Biotechnologies and health Sciences, University of Torino, Torino, Italy.

^2^ Department of Molecular Biotechnologies and health Sciences, University of Torino, Torino, Italy.

^3^ Department of Medicine and Aging Science, Center of Excellence on Aging and Translational Medicine (CeSi-Met), G. D’Annunzio University, Chieti-Pescara, Italy

*Dr. Giuseppe Ferrauto, giuseppe.ferrauto@unito.it; Molecular Imaging Center, Department of Molecular Biotechnologies and health Sciences, University of Torino, Via Nizza 52, 10126 Torino (It)- Tel: +390116708459

^†^ These authors equally contributed to the work

The authors declare no potential conflicts of interest

Supplementary Materials:

- **Supporting figures**

**Fig.S1 (A) Experimental set-up for MR imaging characterization of mice and validation of results; (B) Representative scheme of anatomy of mice prostate; (C) Representative axial T2w-MR images of healthy C57BL/6 (*left*) and middle stage TRAMP mouse (*right*). Yellow ROIs indicate prostate region.**

**Fig.S2 Labeling of RBCs with Gd-complexex by using hypotonic swelling;**

**Fig.S3 Vascular Volume maps by administration of Gd-HPDO3A-RBCs;**

**Fig.S4 Relative oxygenation maps by administration of Gd-DOTP-RBCs;**

**Fig.S5. PAI signal at 812 nm and SO_2_ assessment by PAI;**

**Fig.S6. Amide Proton Transfer (APT) map of TRAMP mice at three tumor stages and control healthy mouse;**

**Fig.S7. DW images and ADC maps.**

- **Supporting video**

**Video.S1. Multislice axial T_2w_ of representative healthy C57 mice;**

**Video.S2. Multislice coronal T_2w_ of representative healthy C57 mice;**

**Video.S3. Multislice sagittal T_2w_ of representative healthy C57 mice;**

**Video.S4. Multislice axial T_2w_ of representative early stage TRAMP mice;**

**Video.S5. Multislice coronal T_2w_ of representative early stage TRAMP mice;**

**Video.S6. Multislice sagittal T_2w_ of representative early stage TRAMP mice;**

**Video.S7. Multislice axial T_2w_ of representative middle stage TRAMP mice;**

**Video.S8. Multislice coronal T_2w_ of representative middle stage TRAMP mice;**

**Video.S9. Multislice sagittal T_2w_ of representative middle stage TRAMP mice;**

**Video.S10. Multislice axial T_2w_ of representative late stage TRAMP mice;**

**Video.S11. Multislice coronal T_2w_ of representative late stage TRAMP mice;**

**Video.S12. Multislice sagittal T_2w_ of representative late stage TRAMP mice;**

**Video.S13. Multislice axial T_2w_ of representative undifferentiated tumor in late stage TRAMP mice;**

**Video.S14. Multislice coronal T_2w_ of representative undifferentiated tumor in late stage TRAMP mice;**

**Video.S15. Multislice sagittal T_2w_ of representative undifferentiated tumor in late stage TRAMP mice.**

**Supporting figures**


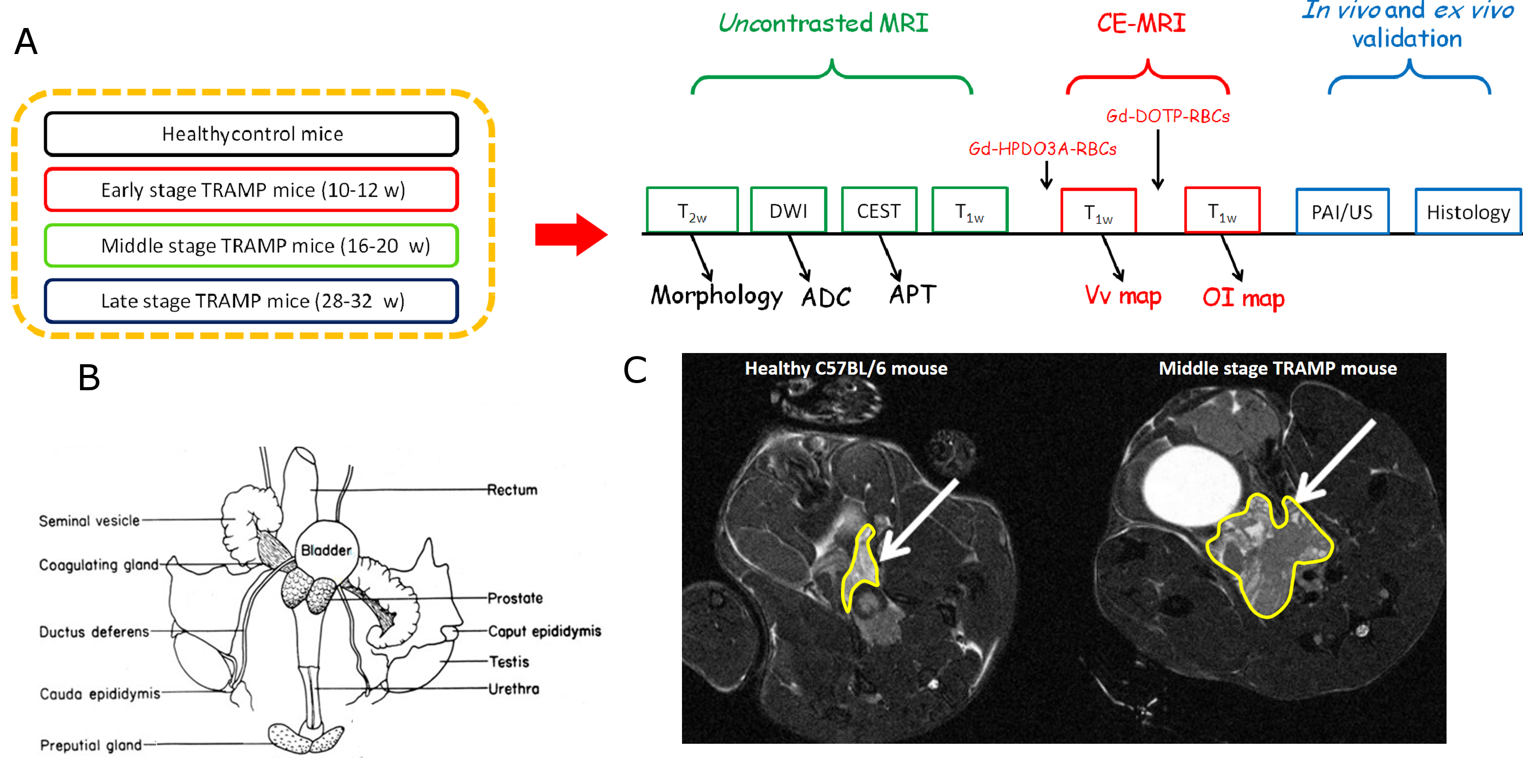


Fig.S1 (A) Experimental set-up for MR imaging characterization of mice and validation of results; (B) Representative scheme of anatomy of mice prostate; (C) Representative axial T_~~2w~~_-MR images of healthy C57BL/6 (*left*) and middle stage TRAMP mouse (*right*). Yellow ROIs indicate prostate region.


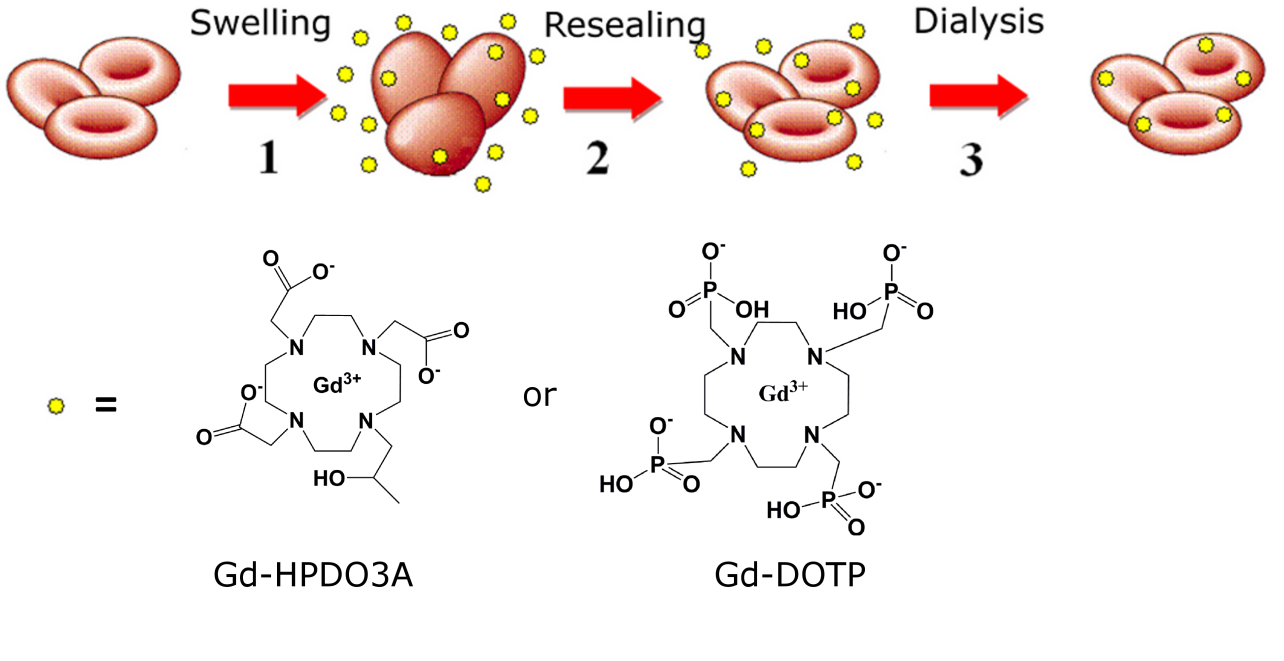


Fig.S2 Labeling of RBCs with Gd-complexex by using hypotonic swelling

**
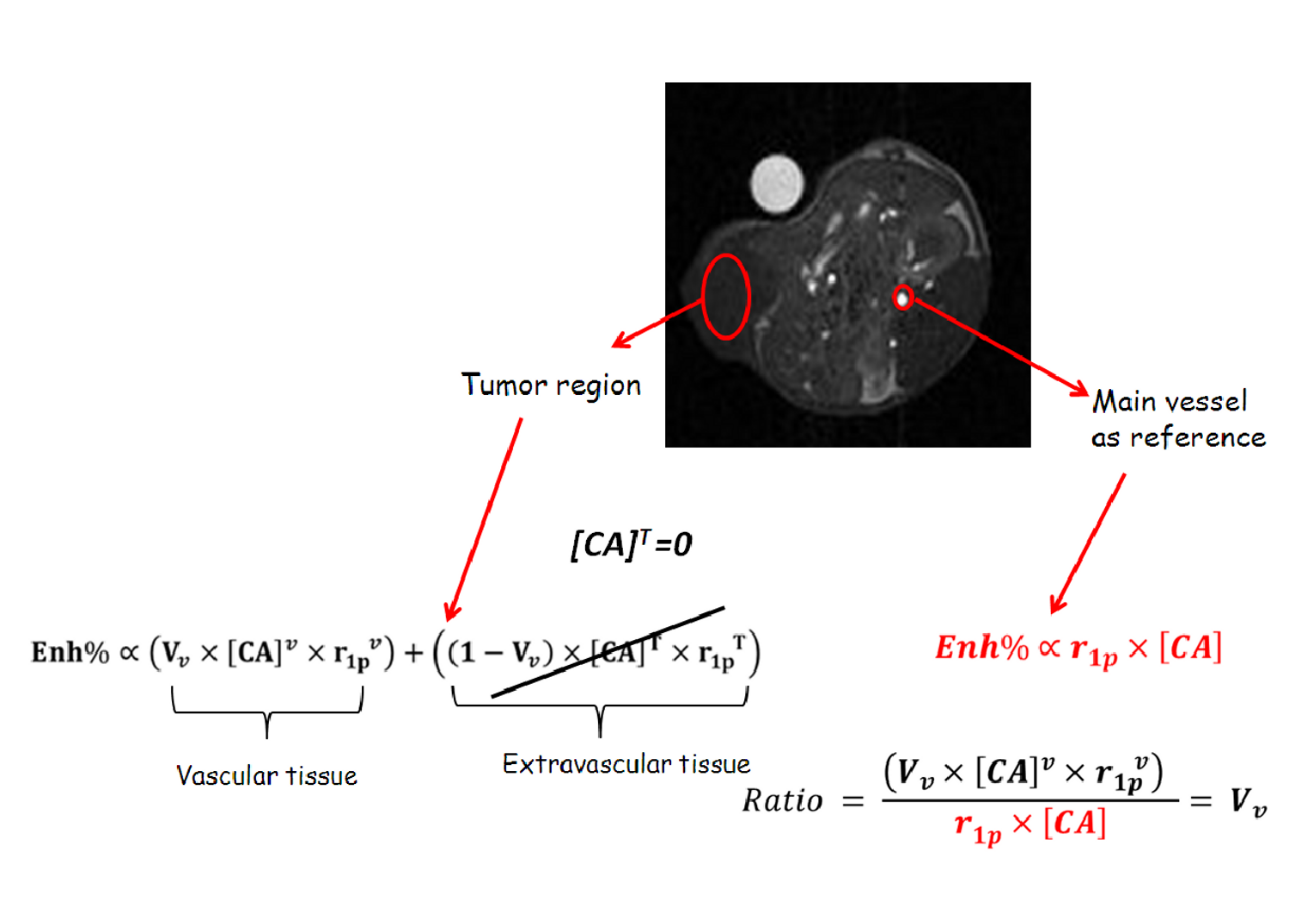
**

Fig.S3 Assessment of Vascular Volume maps by administration of Gd-HPDO3A-RBCs


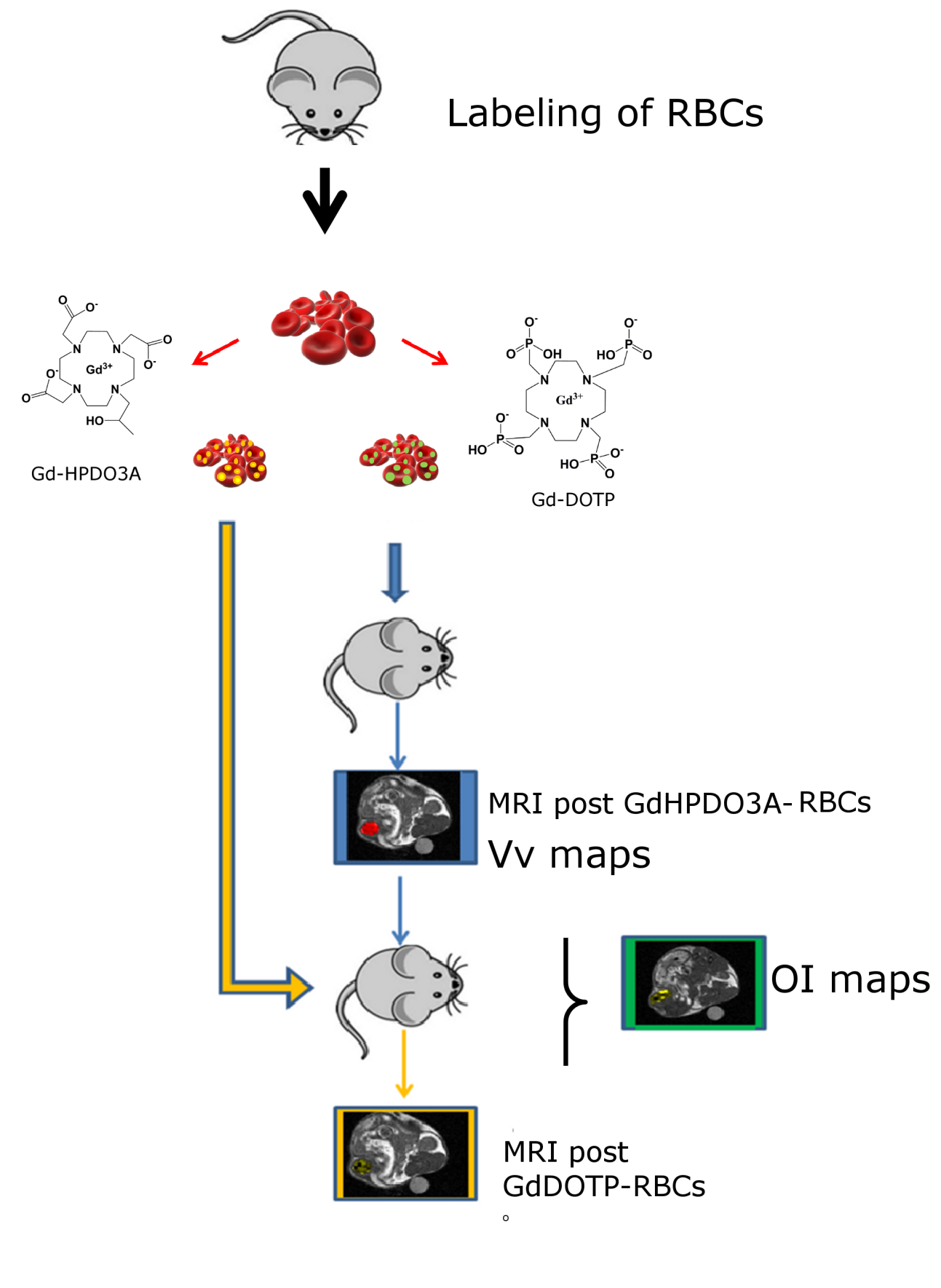


Fig.S4 Relative oxygenation maps by administration of Gd-DOTP-RBCs

**
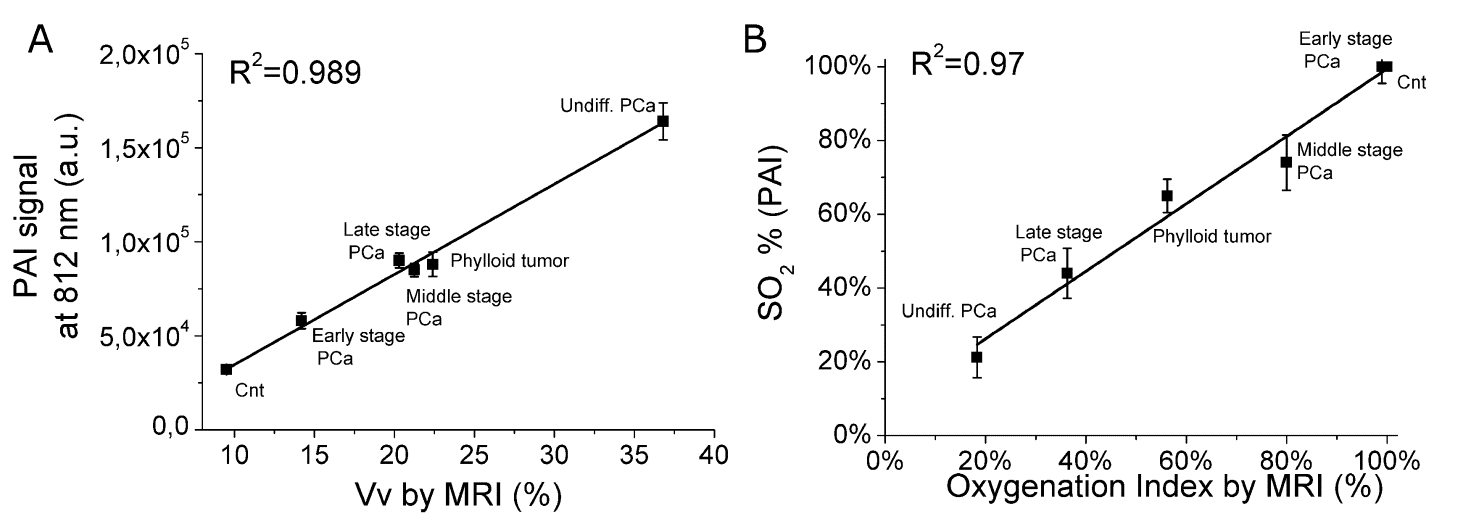
**

Fig.S5. Correlation between (A) PAI signal at 812 nm with Vv assessed by MRI and (B) SO_2_ assessed by PAI with OI assessed by MRI.


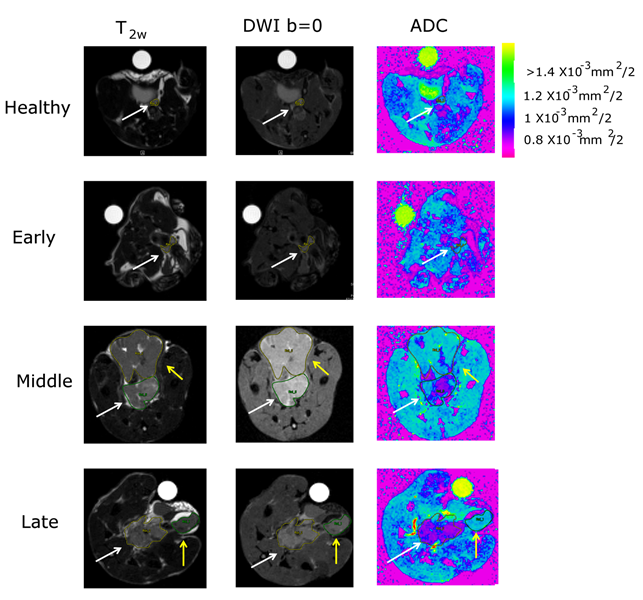


Fig.S6. DW images and ADC maps. T_2w_ image (*left column*), DWI image with b=0 mm^2^/s (*middle column*) and ADC map (*right column*) of control healthy C57BL/6 mice (*first line*) and TRAMP mice at early (*second line*), middle (*third line*) and late (*fourth line*) stage*. (White arrow indicates PCa; yellow arrow indicates seminal vesicles phyllodes tumor).*


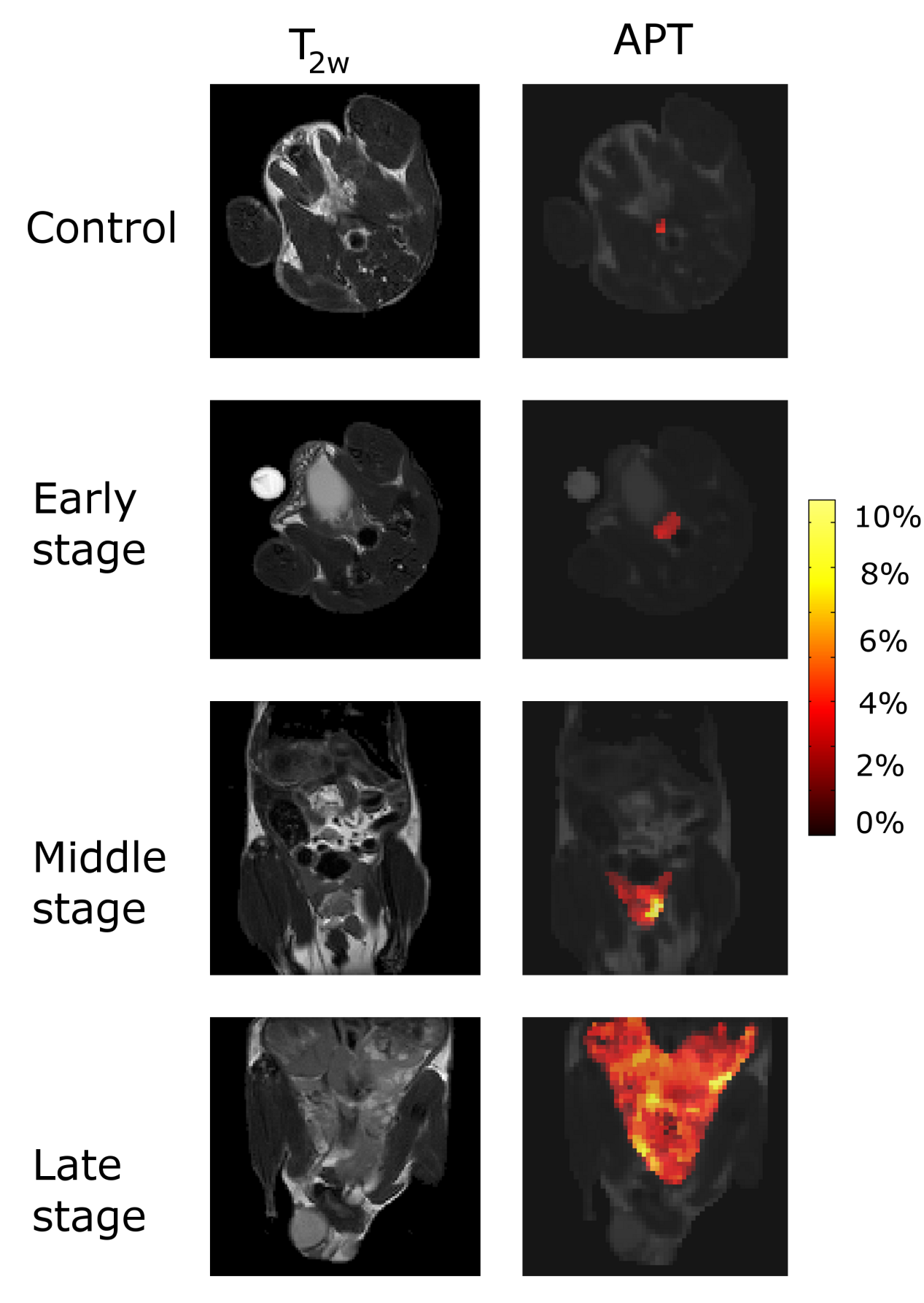


Fig.S7. Amide Proton Transfer (APT) map of TRAMP mice at three tumor stages and control healthy mouse.
